# Of Commensals and Opportunists: Genomics of Coagulase‐Negative Staphylococci During Sequential Ear and Eye Infections in a Healthy Adult

**DOI:** 10.1002/mbo3.70277

**Published:** 2026-03-31

**Authors:** Soo Sum Lean, Chew Chieng Yeo, Zain Illyaaseen, Sargit Kaur, Yun Fong Ngeow, Stuart C. Clarke, Hien Fuh Ng

**Affiliations:** ^1^ Faculty of Medicine and Institute for Life Sciences, University of Southampton Southampton UK; ^2^ Centre for Research in Infectious Diseases & Biotechnology (CeRIDB), Faculty of Medicine Universiti Sultan Zainal Abidin Kuala Terengganu Terengganu Malaysia; ^3^ Department of Pre‐Clinical Sciences, M. Kandiah Faculty of Medicine and Health Sciences Universiti Tunku Abdul Rahman Kajang Selangor Malaysia; ^4^ Department of Biological Sciences, Faculty of Science Universiti Tunku Abdul Rahman Kampar Perak Malaysia; ^5^ Institute for Research, Development and Innovation International Medical University Kuala Lumpur Malaysia; ^6^ NIHR Southampton Biomedical Research Centre University Hospital Southampton Foundation NHS Trust Southampton UK

**Keywords:** coagulase‐negative staphylococci, community‐associated, SCC*mec*, *Staphylococcus capitis*, *Staphylococcus epidermidis*, whole‐genome sequencing

## Abstract

Coagulase‐negative staphylococci (CoNS) are ubiquitous skin commensals but can cause opportunistic infections and may serve as reservoirs for mobile genetic elements such as the staphylococcal cassette chromosome *mec* (SCC*mec*) associated with healthcare‐adapted lineages. In this longitudinal, single‐participant case study, we characterized CoNS isolates obtained over time from a healthy 32‐year‐old male who experienced two infection episodes 6 months apart (left ear, followed by the left eye), together with post‐recovery sampling from additional body sites. Six CoNS isolates were recovered, tested for antimicrobial susceptibility and subjected to whole‐genome sequencing. Genome‐based identification showed that the ear and arm isolates were *Staphylococcus capitis* (subsp. *urealyticus* and subsp. *capitis*), whereas three isolates from the symptomatic left eye were *Staphylococcus epidermidis*. The three *S. capitis* isolates belonged to distinct sequence types (ST1, ST2, and ST10), while the three *S. epidermidis* isolates shared a novel sequence type, ST1284. A non‐typable, mosaic SCC*mec* type IV element homologous to SCC*mec* IVa/IVn was identified in the multidrug‐resistant *S. capitis* subsp. *urealyticus* ST1 isolate E_e2, recovered from the infected ear. Core‐genome phylogeny and genomic signatures placed E_e2 within the proto‐NRCS‐A clade, indicating that a lineage closely related to hospital‐associated outbreak strains can be encountered in a community‐dwelling individual without known prior healthcare exposure. These findings provide a within‐host genomic snapshot of CoNS dynamics across contiguous anatomical sites, and while the study does not establish causality for infection or permit population‐level inference, it highlights the need for broader, systematic community surveillance of CoNS lineages and their mobile genetic elements.

## Graphical abstract

The genomes of coagulase‐negative staphylococci (CoNS) isolated during sequential ear and eye infections in a healthy adult are analyzed. We highlight the identification of a community‐acquired *S. capitis* subsp. *urealyticus* ST1 lineage carrying a mosaic SCC*mec* IV element, linking common skin commensals to multidrug‐resistant strains associated with hospital neonatal outbreaks.

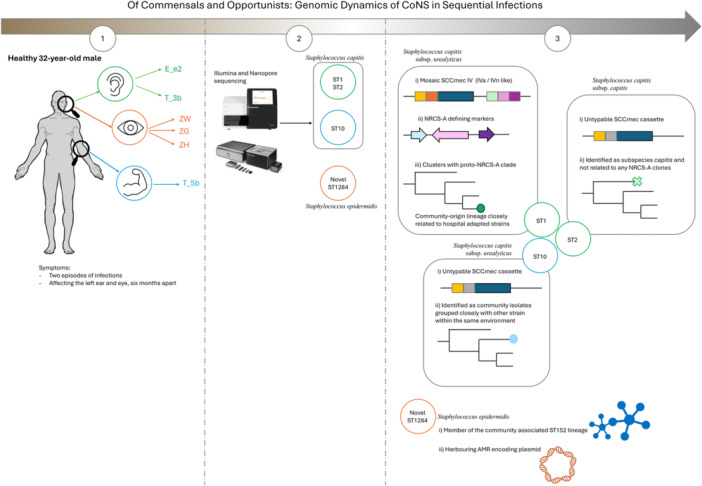

## 1 Introduction

The skin surface, in particular the epidermis, constitutes the human body's first line of defense, forming a physical barrier against harmful microbes. As the largest organ that covers approximately 25 m^2^, the skin is home to a diverse community of commensal microorganisms (Boxberger et al. 2021). Members of the genera *Staphylococcus*, *Corynebacterium*, and *Streptococcus* are among the commonly detected taxa (Parlet et al. 2019). This barrier extends to anatomically contiguous sites such as the external auditory canal and eyelid margins, while the ocular surface itself hosts a low‐biomass but distinct microbiota in which *Staphylococcus*, *Streptococcus* and related genera are frequently detected (Petrillo et al. 2020; Flores‐Páez et al. 2015). Within these cutaneous and peri‐mucosal niches, *Staphylococcus* is one of the most frequently isolated genera (Parlet et al. 2019). Staphylococci can be broadly divided into coagulase‐positive staphylococci (CoPS) and coagulase‐negative staphylococci (CoNS) with CoPS being best exemplified by the major pathogen *Staphylococcus aureus* (Becker et al. 2014). Although *S. aureus* can cause severe skin infections, particularly in immunocompromised patients, as well as at ocular sites (Chiang and Chern 2022), it is outnumbered on healthy skin and related surfaces by CoNS (Parlet et al. 2019). CoNS communities appear to outcompete *S. aureus* through the production of a diverse arsenal of antimicrobial compounds (Janek et al. 2016). Thus, CoNS are better adapted to the harsh conditions of the skin surfaces including lower temperatures, acidity and nutrient limitation (Parlet et al. 2019; Boxberger et al. 2021).

Although the composition of CoNS varies across body sites, species such as *S. epidermidis*, *S. hominis*, *S. capitis*, *S. haemolyticus* and *S. saprophyticus* are generally observed on the skin (Cavanagh et al. 2016; Parlet et al. 2019). Similar CoNS species are also recovered from the external auditory canal and ocular surfaces where they are frequently reported as commensals but can also act as opportunistic pathogens in otitis media/externa and superficial ocular infections such as conjunctivitis and keratitis (Flores‐Páez et al. 2015; Kim and Yeo 2017; Petrillo et al. 2020; Chiang and Chern 2022). Historically, CoNS have been regarded as non‐dominant staphylococci and largely harmless commensals, and consequently often escaped rigorous pathogen surveillance. This underestimation has led to the surge of CoNS infections in vulnerable settings such as the occurrence of several outbreaks of the *S. capitis* NRCS‐A clone in neonatal intensive care units (NICUs) (Laurent and Butin 2019; Hilmarsdóttir et al. 2025). The global spread of *S. capitis* has redefined this species as a significant pathogen, in particular those categorized as subsp. *urealyticus* of the National Reference Center for Staphylococci‐A (NRCS‐A) lineage, while it continues to exist as a commensal in healthy communities (Wirth et al. 2020; Pinheiro‐Hubinger et al. 2021; Hilmarsdóttir et al. 2025). In the neonatal healthcare settings, *S. capitis* has been shown to be more life‐threatening than other CoNS (Isaacs 2003). The emergence of *S. capitis* as a neonatal pathogen is closely associated with late‐onset sepsis (LOS), which accounts for 20%–30% of infections among preterm neonates in NICUs (Stoll et al. 2010; Laurent and Butin 2019). Eradication of *S. capitis* remains challenging in healthcare settings, as the pathogen readily colonizes abiotic surfaces, including incubators and indwelling medical devices, and also exhibits reduced susceptibility to antiseptics (Laurent and Butin 2019; Felgate et al. 2023).

*Staphylococcus epidermidis* is another human skin commensal that is frequently implicated in medical device colonization and infection, especially in neonatal and immunocompromised patients (Thomas et al. 2014; Datta et al. 2021). Similar to *S. capitis*, the pathogenic potential of *S. epidermidis* was long underestimated because of its status as a core commensal microbiota. However, reports of *S. epidermidis* responsible for 35% of monomicrobial prosthetic joint infections (PJI) and 59% of polymicrobial PJI (Flurin et al. 2019) underscores its importance as a serious opportunistic pathogen. Beyond implant‐associated infections, *S. epidermidis* is also recognized as a causal agent of several ocular infections including keratitis, conjunctivitis and endophthalmitis, highlighting its opportunistic behavior on the eye surface (Flores‐Páez et al. 2015; Petrillo et al. 2020). The “accidental” pathogenic success of *S. epidermidis* lies in its genomic plasticity, which enables rapid acquisition of antimicrobial resistance genes along with virulence traits such as biofilm formation (Both et al. 2021; Datta et al. 2021). The adaptability of *S. epidermidis* in healthcare settings is further exemplified by the global dissemination of sequence type 2 (ST2), a lineage characterized by antimicrobial resistance and robust biofilm production (Miragaia et al. 2007; Lee et al. 2018; Both et al. 2021). As more *S. epidermidis* lineages are identified as hospital‐adapted (Thomas et al. 2014; Lee et al. 2018), it becomes a pressing matter to understand the diverse lifestyle and persistence strategies of this species across different anatomical sites.

The skin and its contiguous structures, including the external auditory canal and peri‐ocular skin, thus represent one of the most diverse reservoirs of CoNS, yet the composition and distribution of CoNS across these body sites remain poorly understood. While strain diversification of *S. epidermidis* in infants has been demonstrated (Datta et al. 2021), data on other CoNS species in healthy adults are still limited. Lately, healthcare settings are increasingly affected by CoNS infections (Lee et al. 2018; Wirth et al. 2020; Wan et al. 2023), but the routes by which these strains disseminate from the community to the hospital environment are unclear. Although it has been proposed that CoNS may originate from community sources such as healthcare personnel or pre‐colonized patients (Michels et al. 2021), strain selection and adaptation within specific microenvironments such as the ear canal and ocular surface, remain largely understudied.

Here, we investigate the genomic features of *S. capitis* and *S. epidermidis* strains isolated from a healthy individual who developed sequential ear and eye infections. By characterizing these isolates and comparing their distribution across various body sites, we aim to capture shifts in CoNS species and strains, providing a genomic snapshot of potential links between community‐ and hospital‐colonizing CoNS.

## 2 Methodology

### 2.1 Study Individual and Symptom Descriptions

A 32‐year‐old male experienced two distinct infection episodes occurring 6 months apart, affecting the left ear and subsequently the left eye. During the first episode, the individual reported that his left ear felt moist, congested, and heavy. A discharge sample from the ear canal was collected for microbiological analysis. From the primary culture, one colony representing the predominant colony morphotype was selected for downstream purification and characterization; this colony was designated E_e2. The individual was subsequently treated with trimethoprim‐sulfamethoxazole due to his allergy to penicillin. Follow‐up sampling was performed 88 days (~3 months) after the initial sampling and corresponded to the individual's follow‐up visit. This time point was intended to reduce short‐term antimicrobial perturbation effects and allow re‐establishment of commensal flora. Swab samples were collected from various body sites (scalp, ear canals, neck, arms, and legs) of the individual. Two samples, designated T_3b and T_5b, were recovered from the left ear and left arm (at the cubital fossa), respectively.

Six months after the ear infection, the same individual developed ocular symptoms involving the left lower eyelid which appeared slightly swollen, producing thick and watery eye discharge differing from normal tears. He also reported discomfort in contact lens use during this occurrence. A swab sample was obtained from the red granulation tissue by gently pulling down the lower eyelid. Three isolates, namely ZG, ZW, and ZH, were recovered from this swab sample. The individual was prescribed chloramphenicol eye drops for this infection.

### 2.2 Bacterial Isolation, Preliminary Identification, and Antimicrobial Susceptibilities

All swab samples were inoculated onto sheep blood agar (Isolab, Malaysia) and incubated at 37°C for 24–48 h. Subsequently, colonies were sub‐cultured and purified on sheep blood agar for further identification. For the ear discharge sample, colonies with the predominant morphotype were prioritized for selection. Minor morphotypes, where present, were not retained for further characterization. Gram staining was carried out to identify morphologies of the purified bacterial colonies. These isolates were then subjected to 16S rDNA PCR‐Sanger sequencing for preliminary identification as described in Lean et al. (2025). Antimicrobial susceptibilities were determined using the disk diffusion (Oxoid, UK) and the BD Phoenix^TM^ M50 system's PMIC‐GPC panel (Becton, Dickinson and Company, USA).

### 2.3 Whole Genome Sequencing

Total DNA samples were extracted from the six identified isolates and short‐read Illumina sequencing was performed as described previously (Lean et al. 2025). Meanwhile, long‐read sequencing was performed on one of the isolates (designated E_e2) using the Oxford Nanopore Technologies (ONT) platform. A total of 500 ng of total DNA, quantified with the Qubit High Sensitivity dsDNA Assay (Thermo Fisher Scientific, Waltham, MA, USA), was used as input for library preparation with the Ligation Sequencing Kit LSK114 (ONT), following the manufacturer's instructions. Sequencing was conducted on a Flongle R10.4.1 flow cell attached to a MinION Mk1B sequencer (ONT), retaining only raw signal data with predicted read lengths exceeding 1,000 bp. Basecalling of the POD5 files was performed using Dorado v0.7.0 (available from https://github.com/nanoporetech/dorado) with the simplex dna_r10.4.1_e8.2_400bps_sup_v5.0.0 model.

### 2.4 Sequence Quality Assessments and Genome Assemblies

Short‐read sequences (2 × 150 bp) of the six isolates were quality assessed and filtered from poor quality reads via fastP (Chen et al. 2018). The quality of the long‐read sequences was checked and filtered (*q* ≥ 10) using Nanoplot and Nanofilt, respectively. Filtered short‐ and long‐ reads were subjected to (i) short‐read assembly, (ii) hybrid assembly and (iii) long‐read assembly using different assemblers. For (i) and (ii), genome assemblies were carried out using the Unicycler pipeline (Wick et al. 2017) whereas Autocycler (Wick et al. 2025) was applied to the long‐read assembly. Secondary quality check using CheckM (Parks et al. 2015) was applied to all genome assemblies to detect contamination and evaluate their completeness.

### 2.5 Bioinformatics Analysis

Resulting genome assemblies were subjected to species identification using Kraken2 (Wood and Salzberg 2014) and fastANI, followed by multilocus sequence type (MLST) determination via mlst software. MLST profiles of the genomes were also cross‐checked with existing sequence types (STs) in PubMLST (https://pubmlst.org/). Genome annotation was performed using PROKKA 1.14.6 (Seemann 2014) and Bakta v1.11.4 (https://github.com/oschwengers/bakta). The genomes were also screened for Staphylococcal Chromosome Cassette *mec* (SCC*mec*) using staphopia‐sccmec (https://github.com/staphopia/staphopia-sccmec) and sccmec (https://github.com/rpetit3/sccmec) tools. Novel and/or unusual SCC*mec* regions were further investigated through manual BLAST. Genotypic antimicrobial resistance (AMR) of the genomes was determined through AMRFinderPlus (Feldgarden et al. 2021) and Abricate, utilizing databases from NCBI, Resfinder (Bortolaia et al. 2020) and CARD (Alcock et al. 2023). Virulomes were determined using VFAnalyzer, in addition to the —plus function from AMRFinderPlus and virulence factor database (VFDB) (Liu et al. 2022) utilized in Abricate. Unique virulence factors (VFs) were identified using manual BLAST, according to relevant published sources. Defense systems and phages embedded within the genomes were identified using defense‐finder and PHASTER webtool (available from https://phaster.ca/), respectively. Pangenome analysis was performed using Panaroo (Tonkin‐Hill et al. 2020), alongside reference genomes obtained from NCBI and ENA databases. Phylogenetic trees were constructed using VeryFastTree (Piñeiro et al. 2020) and visualized in iTOL v7 (Letunic and Bork 2024). Graphical visualization of the genomes and mobile genetic elements (MGEs) were carried out using Bandage (Wick et al. 2015) and EasyFig (Sullivan et al. 2011), respectively. Graphs and charts were plotted using R packages available in RStudio version 2023.06.0 + 421.

### 2.6 Ethical Approval

Informed consent was obtained from the patient, and the study was approved by the UTAR Scientific and Ethical Review Committee (U/SERC/78‐297/2024). All experiments were performed in accordance with relevant guidelines and regulations.

## 3 Results and Discussions

### 3.1 Presumptive CoNS Isolation

Initial swab sample from the infected left ear of the otherwise healthy individual resulted in the isolation of the presumptive staphylococcal strain designated E_e2, which showed hemolysis on sheep blood agar. The individual was prescribed a 10‐day course of oral trimethoprim‐sulfamethoxazole which resolved the ear infection. Eighty‐eight days after the infection, swabs were taken from the left ear as well as other parts of the individual's body (as indicated in the Methods) corresponding to the individual's follow‐up visit. Two presumptive staphylococcal strains on sheep blood agar were obtained from the left ear (designated T_3b) and the cubital fossa of the left arm (designated T_5b). All three strains (i.e., E_e2, T_3b, and T_5b) were Gram‐positive cocci and preliminary identification via 16S rDNA PCR‐sequencing analysis revealed them as *S. capitis*/*S. caprae*. The same individual returned with a left eye infection 6 months later, and three presumptive staphylococci were recovered from a single swab of the infected eye. These additional three isolates were designated ZG, ZH, and ZW. These isolates were similarly Gram‐positive cocci but preliminary species identification through 16S rDNA sequencing showed them as *S. epidermidis*.

### 3.2 Preliminary Genome Analysis

Whole genome sequencing of the six presumptive staphylococcal strains (i.e., E_e2, T_3b, T_5b, ZG, ZH, and ZW) resulted in assembled genome sizes ranging from ~2.41 Mb to ~2.55 Mb (Table 1). Among the genomes, strain T_5b was found to harbor the highest number of plasmids (*n* = 3), while T_3b harbored only one plasmid. E_e2 was found to carry two plasmids (Table 1) and was also subjected to long‐read sequencing using the ONT platform. The three strains that originated from the infected left eye (i.e., ZG, ZH, and ZW) all harbored two plasmids, as shown in Table 1. Basic statistics of the genome assemblies were as listed in Table 1.

**Table 1:** Strains obtained from the infected individual and their preliminary genomic information.

	Short‐read assembly						Hybrid assembly	Long‐read assembly
	E_e2	T_3b	T_5b	ZG	ZH	ZW	E_e2	E_e2
Isolation site	Left ear	Left ear	Left arm	Left eye	Left eye	Left eye	Left ear	Left ear
Species	*S. capitis*	*S. capitis*	*S. capitis*	*S. epidermidis*	*S. epidermidis*	*S. epidermidis*	*S. capitis*	*S. capitis*
MLST	ST1	ST2	ST10	ST1284	ST1284	ST1284	ST1	ST1
SCC*mec* type	IVa/IVn	NA	NA	NA	NA	NA	IVa/IVn	IVa/IVn
Total reads	9,913,492	9,803,118	9,800,682	12,724,750	10,838,248	12,122,260	514,515,499	504,602,007
GC content (%)	33.29	33.28	32.94	32.37	32.76	32.25	33.02	33.02
Contigs (*n*)	65	55	90	84	79	79	3	3
Size (bp)	2,522,730	2,415,952	2,488,172	2,428,418	2,428,575	2,428,227	2,554,961	2,554,960
N50 (bp)	557,042	697,389	1,284,241	138,739	138,740	138,740	2,546,540	2,546,540
Completeness (%)	99.81	99.81	99.81	99.78	99.78	99.78	99.81	99.81
Contamination (%)	0.36	0.06	0.08	0	0	0	0.36	0.36
Plasmids (*n*)	(2) p1‐E_e2 (2473 bp) p2‐E_e2 (5,948 bp)	(1) p1‐T_3b (3594 bp)	(3) p1‐T_5b (8387 bp) p2‐T_5b (31,185 bp) p3‐T_5b (67,531 bp)	(2) p1‐ZG (1318 bp) p2‐ZG (17,387 bp)	(2) p1‐ZH (1318 bp) p2‐ZH (17,737 bp)	(2) p1‐ZW (1318 bp) p2‐ZW (17,387 bp)	(2) p1‐E_e2 (2473 bp) p2‐E_e2 (5,948 bp)	(2) p1‐E_e2 (2473 bp) p2‐E_e2 (5,948 bp)

**Figure 1:**
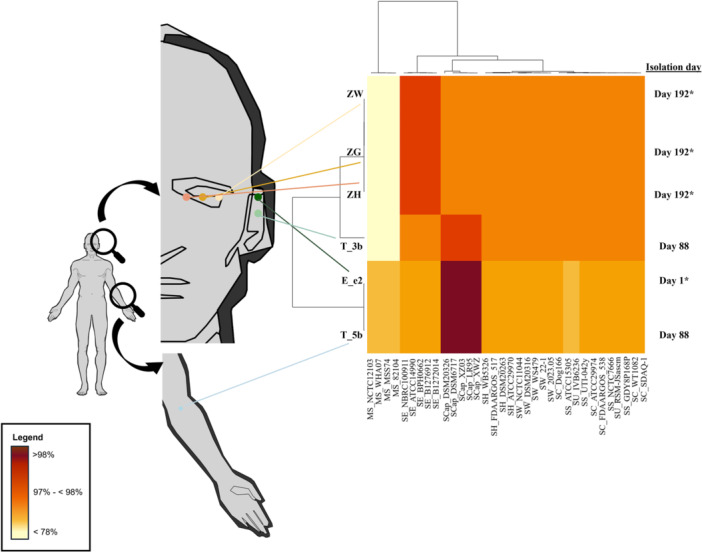
The origin of the CoNS strains in this study and their average nucleotide identity (ANI) values. Anatomical diagrams on the left indicate swab sites from which the CoNS strains were isolated, while the heatmap on the right shows the corresponding ANI values for the genomes of the six CoNS that were sequenced. An asterisk (*) in the isolation day column denotes strains isolated prior to any antibiotic treatment. Abbreviations used: *Mammaliicoccus sciuri* (MS), *Staphylococcus epidermidis* (SE), *Staphylococcus capitis* (SCap), *Staphylococcus haemolyticus* (SH), *Staphylococcus warneri* (SW), *Staphylococcus cohnii* (SC), *Staphylococcus saprophyticus* (SS), and *Staphylococcus urealyticus* (SU).

Species identification based on average nucleotide identity (ANI) validated strains E_e2, T_3b, and T_5b as *S. capitis*, with > 98% homology to *S. capitis* reference genomes in Figure 1. Detailed identification revealed strains E_e2 and T_5b as *S. capitis* subsp. *urealyticus*, whereas strain T_3b was identified as *S. capitis* subsp. *capitis*. Strains ZG, ZH, and ZW were identified as *S. epidermidis*, based on the homology shown in Figure 1. Hence, these findings validated the presence of two CoNS species from two different body sites during the course of different infections in the same individual. It is apparent that the left ear and left arm of the infected individual were dominated by *S. capitis*, whereas the left eye was dominated by *S. epidermidis* (Figure 1).

The three *S. capitis* strains were each of different STs, with E_e2 being ST1 [of Clonal Complex 1 (CC1)], T_3b was typed as ST2 (CC2), and T_5b was ST10 (CC1), based on the MLST scheme recently proposed by Wang et al. (2025). In the case of the *S. epidermidis* strains, all three were of the same novel ST and which was assigned as ST1284 [*arc*(1)‐*aroE*(1)‐*gtr*(2)‐*mutS*(6)‐*pyrR*(2)‐*tpiA*(16)‐*yqiL*(86)] (Table 1) by the PubMLST curators.

### 3.3 Novel SCC*mec* Cassette Discovered From the Community‐Associated *S. capitis* subsp. *urealyticus* Strains

**Figure 2:**
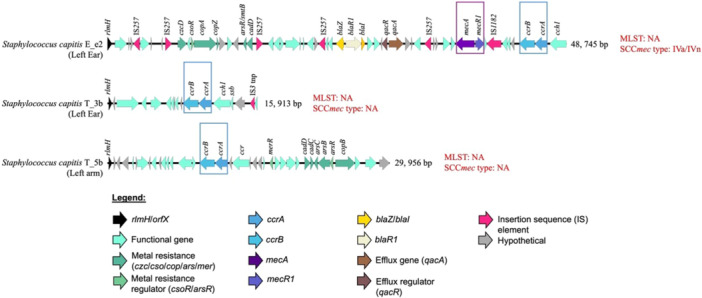
Structural maps of Staphylococcal Chromosome Cassette mec (SCC*mec*) discovered in the genomes of *S. capitis* strains isolated in this study. The hallmark *mec* and *ccr* complexes are bracketed in purple and blue boxes, respectively. Nearest SCC*mec* type identified in each genome is stated in red fonts. Note that in strains T_3b and T_5b, the *mec* genes were absent, and thus these elements can be categorized as ΨSCC elements (Becker et al. 2014). Abbreviations: MLST, multilocus sequence type; NA, not available.

*S. capitis* subsp. *urealyticus* strains E_e2 and T_5b, together with *S. capitis* subsp. *capitis* T_3b, were found to contain SCC elements (Figure 2). Among the three genomes, only the SCC cassette from *S. capitis* subsp. *urealyticus* E_e2 fulfilled the definitions of a *bona fide* SCC*mec* cassette (Uehara 2022; Wolska‐Gębarzewska et al. 2023). Although complete with *mec* and *ccr* complexes (Figure 2), the SCC*mec* cassette from *S. capitis* subsp. *urealyticus* E_e2 displayed unusual combinations of genes and mobile elements. The *mec* complex was composed of the IS*431*‐*mecA*‐Δ*mecR1*‐IS*1272* structure (Figure 2), which classified it as a class B *mec* complex (Wolska‐Gębarzewska et al. 2023). Paired with the presence of *ccrA2* and *ccrB2* within the *ccr* complex (Figure 2), the SCC*mec* cassette from *S. capitis* subsp. *urealyticus* E_e2 was classifiable as a SCC*mec* type IV (Uehara 2022; Wolska‐Gębarzewska et al. 2023). However, further classification of the SCC*mec* type IV depends on the “junkyard” regions (J regions) (Uehara 2022). The J region from the SCC*mec* cassette of strain E_e2 showed combinations from SCC*mec* type IVa and IVn, whereby its J1 region matched to that in SCC*mec* type IVa and its J3 region to SCC*mec* type IVn. These unusual features of the SCC*mec* in E_e2 was validated through obtaining its complete sequence by long‐read sequencing and hybrid assembly (Figure 2). These unusual combinations of genetic elements resulted in homology to multiple subtypes of the currently known SCC*mec* type IV, and thus the SCC*mec* cassette from *S. capitis* subsp. *urealyticus* E_e2 can be deemed as non‐typable. In our previous study (Lean et al. 2025), we reported a novel SCC*mec* element from another community‐isolated *S. capitis* subsp. *urealyticus*. The SCC*mec* cassette from *S. capitis* subsp. *urealyticus* E_e2 presented yet another novel cassette, albeit of a type IV composite element, from a community isolate. Besides *mecA* and the *bla* operon, the SCC*mec* in E_e2 also harbored the *qacAR* antiseptic resistance genes along with several metal resistance genes such as arsenic, cadmium and copper (Figure 2). Previous studies have shown that several *S. capitis* NRCS‐A isolates harbor composite or mosaic SCC*mec*–SCC*ars*/*cad*/*cop* elements (Martins Simões et al. 2013; Carter et al. 2018). However, the composite SCC*mec* element identified in E_e2 is markedly different as it is a type IV composite, whereas the elements reported in *S. capitis* NRCS‐A strains CR‐01 (Martins Simões et al. 2013) and NZ‐SC16875 (Carter et al. 2018) are type V composites. Notably, the SCC*mec* composites in CR‐01 and NZ‐SC16875 also carry a subtype IIIA CRISPR–Cas locus, which is absent in E_e2 (Figure A1).

The SCC cassettes found immediately downstream of the *rlmH/orfX* integration site in *S. capitis* subsp. *capitis* T_3b and *S. capitis* subsp. *urealyticus* T_5b showed entirely different combinations of genetic elements (Figure 2). Both cassettes lacked the *mec* complex but harbored the *ccr* complex, which consists of *ccrA* and *ccrB* (Figure 2). *S. capitis* subsp. *capitis* T_3b exhibited the simplest SCC cassette in this collection, whereas the cassette from *S. capitis* subsp. *urealyticus* T_5b was embedded with heavy metal resistance determinants (Figure 2). Genes responsible for resistance towards metalloids and transition metals with toxic/bactericidal properties, encompassing arsenic (*ars*), cadmium (*cad*), copper (*copB*) and mercury (*mer*) (Becker et al. 2014), were discovered within the SCC cassette from *S. capitis* subsp. *urealyticus* T_5b. Therefore, this cassette was denoted as SCC*ars*‐*cad*‐*cop*‐*mer* and similar compositions in SCC cassettes have also been reported in other staphylococcal species (Kinnevey et al. 2013; Becker et al. 2014). Although untypable, the SCC cassettes from *S. capitis* subsp. *capitis* T_3b and *S. capitis* subsp. *urealyticus* T_5b (Figure 2) can be classified as pseudo (Ψ) SCC elements (Becker et al. 2014), which highlighted the lesser‐studied variation of the SCC family of elements that are without the *mec* complex.

These observations suggest temporal changes in the *S. capitis* strains recovered from this individual during a symptomatic ear episode and at follow‐up after antibiotic treatment. Specifically, the *S. capitis* isolate recovered during the ear infection (subsp. *urealyticus* strain E_e2) differed from the follow‐up ear isolate (subsp. *capitis* strain T_3b) (Figure 1). In parallel, SCC*mec* was detected in E_e2 but not in T_3b (Figure 2). Given that SCC*mec* elements encompass diverse subtypes and are frequently reported among clinical staphylococcal isolates (Becker et al. 2014; Du et al. 2021; Uehara 2022; Hilmarsdóttir et al. 2025), the identification of a composite SCC*mec* type IV element in *S. capitis* E_e2 indicates the potential for clinically relevant mobile genetic elements to occur outside healthcare settings. However, with the current sampling design and single‐colony selection for the symptomatic ear episode, we are unable to infer causality, directionality of acquisition or loss, or that SCC*mec* is required for symptomatic presentation. Broader longitudinal sampling would be needed to resolve these dynamics.

### 3.4 The Community‐Associated CoNS Demonstrated Multidrug Resistance Capability

In the earlier sections, two different CoNS species (i.e., *S. capitis* and *S. epidermidis*) from the healthy individual were described. To further elucidate their differences at interspecies and intraspecies levels, AMR profiles of these isolates were investigated. First, we will describe the differences in their resistomes at interspecies level (i.e., *S. capitis* vs*. S. epidermidis*) and then narrow down to intraspecies level (i.e., *S. capitis* subsp. *capitis* vs*. S. capitis* subsp. *urealyticus*), particularly pre‐ and post‐antibiotic treatment for the latter.

**Figure 3:**
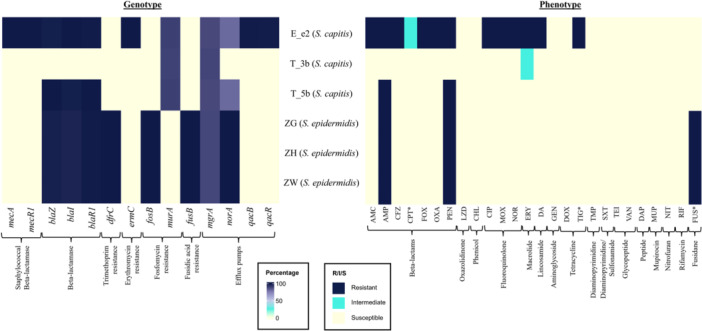
AMR profiles of the six strains in this study, as identified genotypically and observed phenotypically. Heatmap on the left represents resistance determinants identified in the six genomes, marked with percentage identities to the homologous genes in *S. aureus*. Phenotypic AST results are shown on the right, specified with color coded resistance levels. Drug panels used in the AST were abbreviated as listed below, and antibiotics marked with an asterisk (*) indicate that the susceptibility test was only performed using disk diffusion. All other AST results were obtained from the BD Phoenix M50 system. Details of MIC values and zone of inhibition diameters are in Table A1. AMC = Amoxicillin‐Clavulanate, AMP = Ampicillin, CFZ = Cefazolin, CPT = Ceftaroline, FOX = Cefoxitin, OXA = Oxacillin, PEN = Penicillin G, LZD = Linezolid, CHL = Chloramphenicol, CIP = Ciprofloxacin, MOX = Moxifloxacin, NOR = Norfloxacin, ERY = Erythromycin, DA = Clindamycin, GEN = Gentamicin, DOX = Doxycycline, TIG = Tigecycline, SXT, Trimethoprim/sulfamethoxazole, TEI = Teicoplanin, VAN = Vancomycin, DAP = Daptomycin, MUP = Mupirocin, NIT = Nitrofurantoin, RIF = Rifampin, FUS = Fusidic acid.

Figure 3 shows the spectrum of AMR obtained, in which the *S. capitis* strains (E_e2, T_3b, and T_5b) showed a stark difference to the *S. epidermidis* strains (i.e., ZG, ZH, and ZW) in their AMR profiles. However, all three *S. epidermidis* strains displayed uniform genotypic and phenotypic AMR profiles whereby each strain harbored resistance determinants of up to four classes of antimicrobials (Figure 3). The *S. epidermidis* strains carried the *blaZ*‐*blaR1*‐*blaI* operon which was responsible for beta‐lactam resistance (Becker et al. 2014; Espadinha et al. 2019) and was reflected in the phenotypic resistance towards ampicillin and penicillin shown by all three strains (Figure 3). The *S. epidermidis* strains also shared fusidic acid phenotypic resistance (all three had MIC of > 8 µg/mL) through the carriage of the *fusB* gene (Figure 3; Table A1), which encodes the elongation factor G (EF‐G)‐protecting protein that prevents binding of fusidic acid to the ribosome (Becker et al. 2014; Fernandes 2016; Schoenfelder et al. 2017; Espadinha et al. 2019). In contrast, all three *S. capitis* strains were susceptible to fusidic acid (with MIC of ≤ 0.5 µg/mL).

The *fosB* fosfomycin resistance determinant was detected in the three *S. epidermidis* genomes but not in the *S. capitis* strains, which harbored the *murA* gene instead (Figure 3). However, phenotypic fosfomycin susceptibility could not be determined due to the absence of disk diffusion and broth microdilution breakpoints for staphylococci in both CLSI and EUCAST. Previously, fosfomycin susceptibility in staphylococci was determined by the agar dilution method using Mueller‐Hinton agar supplemented with 25 mg/L glucose 6‐phosphate (Chen et al. 2022) but the latest EUCAST recommendations (v16.0, 2026; available at https://www.eucast.org/bacteria/clinical-breakpoints-and-interpretation/clinical-breakpoint-tables/) no longer listed breakpoints for fosfomycin with a note that antimicrobial susceptibility testing is discouraged for fosfomycin.

The three *S. epidermidis* genomes also harbored the *dfrC* gene which mediates resistance towards trimethoprim (Totake et al. 1998). However, all three strains were susceptible to the trimethoprim/sulfamethoxazole combination (Figure 3) due likely to the absence of the *sul* gene which mediates sulfamethoxazole resistance.

Comparatively, the *S. capitis* strains E_e2, T_3b and T_5b displayed a more diverse spectrum of AMR (Figure 3), likely reflecting differences in sampling timepoints and body sites. While there were variations in the AMR profiles between the *S. epidermidis* and *S. capitis* strains, both species carried the *mgrA* gene (Figure 3), a conserved global regulator found across various staphylococci, orchestrating a plethora of virulence and AMR genes (Crepin et al. 2024; Guo et al. 2024). The *norA* gene was found in all three *S. epidermidis* strains and two of the three *S. capitis* strains (it was absent in *S. capitis* T_3b) (Figure 3). Although *norA* encodes an efflux pump associated with fluoroquinolone and biocide resistance (Ferreira et al. 2022), its presence did not consistently predict phenotypic fluoroquinolone resistance in this dataset, which was only recorded for *S. capitis* E_e2 (Figure 3).

Zooming into *S. capitis* intraspecies comparison, differences in AMR profiles are more evident between subspecies *capitis* and *urealyticus*. Figures 1 and 2 have already illustrated the changes in *S. capitis* subspecies pre‐ and post‐antibiotic treatment in the individual. Regardless, it is worthwhile to note the multidrug resistant (MDR) characteristic of *S. capitis* subsp. *urealyticus* E_e2 which was isolated during the symptomatic ear episode as compared to the more susceptible profile displayed by *S. capitis* subsp. *capitis* strain T_3b isolated from the same ear following symptomatic resolution (Figure 3).

E_e2 was resistant to most tested beta‐lactams, consistent with the carriage of *mecA*‐*mecR1* and the *bla* operon, and showed only intermediate susceptibility to ceftaroline, a fifth‐generation cephalosporin (Figure 3). Its resistance to erythromycin and clindamycin corresponded with its carriage of *ermC* (Figure 3), a determinant linked to resistance towards macrolides, lincosamides and streptogramin B (MLS_B_) in staphylococci (Nemeghaire et al. 2014). The *ermC* gene in E_e2 resides on a small 2473 bp plasmid, p1‐E_e2, discussed further in the subsection on plasmids. E_e2 also harbored *qacB* with *qacR* (Figure 3), genes associated with tolerance to quaternary ammonium compounds and acriflavine (Chavignon et al. 2022). However, since benzalkonium chloride and acriflavine susceptibility was not tested here, the functional impact of *qacB‐qacR* in *S. capitis* remains unknown.

E_e2 additionally showed phenotypic resistance to tigecycline without identifiable canonical determinants. Common drivers of tigecycline resistance in *S. aureus*, such as *tetL* and *mepA* along with mutation in *rpsJ* that encodes ribosomal protein S10 (Angeles Argudin et al. 2018; Wang et al. 2021), were not detected, pointing to possibly alternative mechanisms. This is clinically relevant given the role of tigecycline as a last‐line agent alongside vancomycin and linezolid, and the rarity of tigecycline resistance in *Staphylococcus* spp. (Wang et al. 2021). However, tigecycline resistance in E_e2 was observed only by disk diffusion and could not be confirmed on the BD Phoenix M50 (as tigecycline is not included in the PMIC‐GPC panel). Thus, tigecycline resistance in *S. capitis* E_e2 is unresolved and requires validation by broth microdilution.

By contrast, *S. capitis* subsp. *capitis* strain T_3b, isolated from the patient's left ear 88 days post‐infection, was susceptible to all the tested antimicrobials except for erythromycin where it displayed intermediate susceptibility (MIC of 1 μg/mL) (Figure 3). Concurrently, *S. capitis* subsp. *urealyticus* T_5b, which was isolated from the left arm, was resistant to ampicillin and penicillin but susceptible to erythromycin (with an MIC of 0.5 μg/mL) and all other tested antibiotics. The beta‐lactam resistance in T_5b is explained by the presence of the complete *bla* operon, which was absent in T_3b. However, the mechanism driving the intermediate erythromycin susceptibility in T_3b remains unresolved given the absence of *ermC* in both strains and the shared presence of *mgrA*.

### 3.5 Virulome Profiles Reveal the Presence of the Pathogenic *S. capitis* NRCS‐A Clone

To the best of our knowledge, only Cameron et al. (2015) has reported a comparison of VFs between *S. capitis* subsp. *capitis* and *S. epidermidis* originated from bloodstream infection of the same patient. Scarce data is available on the VFs of these two CoNS species, particularly *S. capitis* subsp. *urealyticus* from the community.

**Figure 4:**
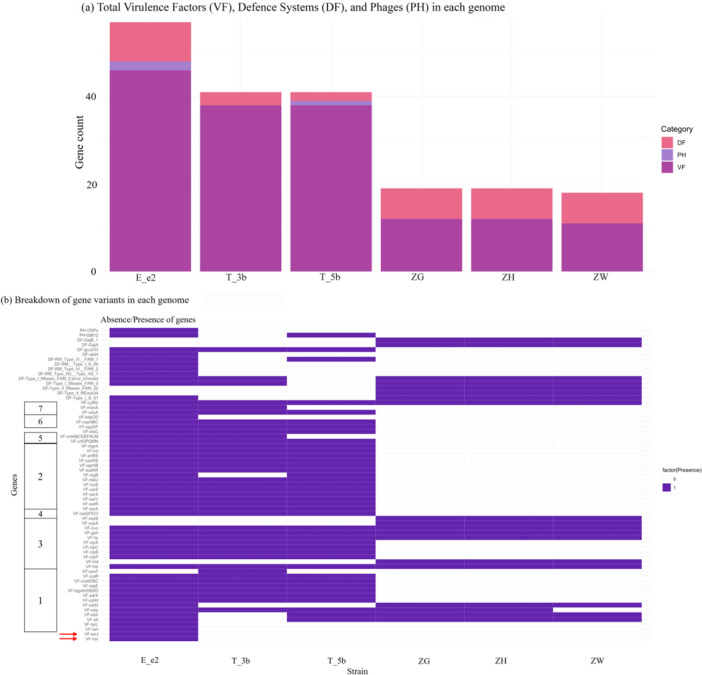
Two‐dimensional plot indicating the total number of related genes present (a) and breakdown of gene variants (b) in each CoNS genome. Gene variants were marked with the abbreviation VF, DF, and PH, representing virulence factor, defense system, and phage, respectively. Boxed numbers on the left represent VF categories; 1 = adherence/surface proteins, 2 = gene regulation, 3 = exoenzymes, 4 = iron acquisition, 5 = ABC permeases, 6 = host immune invasion, 7 = others. Red arrow‐pointed genes were marker genes crucial to the definition of NRCS‐A and proto groups. A high‐definition copy of this diagram is available at https://doi.org/10.6084/m9.figshare.31429514.

Figure 4 showed the differences in the virulomes between *S. capitis* subsp. *capitis* T_3b and *S. capitis* subsp. *urealyticus* E_e2 and T_5b, as well as the distinct variation unique to the *S. epidermidis* strains in this study. The three *S. epidermidis* genomes (i.e., ZG, ZH and ZW) contained fewer VFs as compared to the *S. capitis* strains, with ZG and ZH harboring 11 VFs, whilst ZW contained only ten VFs (Figure 4). The *S. epidermidis* virulomes consisted of surface associated proteins (*atl*, *ebh*, *ebp* and *sdrD*), exoenzymes (*hlb*, *lip*, *geh*, *nuc*, *sspA* and *sspB*) and pro‐inflammatory peptides (*hld*) (Figure 4). The virulomes of the three *S. epidermidis* strains were almost identical with the *ebp* gene only absent in strain ZW (Figure 4). Most of these VFs were cell wall‐related and played a vital role in the formation of biofilms, and facilitating adherence of *S. epidermidis* on (a)biotic surfaces (Cameron et al. 2015; Foster 2020). The exoenzyme VFs were also reported to be involved in biofilm formation and AMR, resulting from their pro‐inflammatory traits (Cameron et al. 2015). The sparse distribution of VFs among the *S. epidermidis* strains was in stark contrast to that displayed in the *S. capitis* strains, whereby their genome contained at least 38 VFs each (Figure 4).

The plethora of VFs present within *S. capitis* subsp. *urealyticus* E_e2 and T_5b, and *S. capitis* subsp. *capitis* T_3b spans across seven categories, possibly influencing the persistence and survival of the bacteria (Figure 4). Strain E_e2 harbored the highest number of VFs (*n* = 46) with T_3b and T_5b having the same number of VFs (*n* = 38 each). Majority of the VFs identified were adherence/surface proteins encoding genes (such as *tarI*, *tarJ*, *tarL*, *atl*, *ebh*, *ebp*, *sdrD*, *sdrH*, *sdrX*, *tagAHGBXD*, *tagE, icaADBC, icaR, sesF*), which function in biofilm formation and/or surface adhesions (Simões et al. 2016; Felgate et al. 2023; Crepin et al. 2024). This is followed by regulatory genes (*sarA*, *sarR*, *sarV*, *sarX*, *luxS*, *rsbU*, *sigB*, *walKR*, *agrAB*, *saeRS*, *arlRS, rot, mgrA*) and VFs encoding exoenzymes (*hlb*, *hld*, *clpP*, *clpB*, *clpC*, *clpX*, *lip*, *geh*, *nuc, sspA*, *sspB*) (Simões et al. 2016; Crepin et al. 2024). Of concern, *S. capitis* subsp. *urealyticus* E_e2 recorded the presence of *tarJ* and the nisin‐resistant determinant *nsr* (Figure 4), which were defining virulence markers for the pathogenic NRCS‐A and proto‐NRCS‐A groups (Felgate et al. 2023). The NRCS‐A clone, in particular, has been implicated in various outbreaks in neonatal intensive care units (NICUs). These two genes, along with the presence of the potassium pump encoding loci *kdpDC*, the *cntABCDEFKLM* operon, *oppDF* loci, and *nikC* gene (Felgate et al. 2023), are suggestive of strain E_e2 being a *S. capitis* NRCS‐A or proto‐NRCS‐A clone. These genes were absent in strains T_3b and T_5b (Figure 4), and are required for the acquisition of metals such as zinc and nickel that are crucial for the pathogenic clone to thrive in the nutrient‐limited environment of the hospital and the human body (Grim et al. 2017). The discovery of these hallmark virulence genes thus indicates strain E_e2 as possibly the sole community‐associated NRCS‐A/proto‐NRCS‐A clone isolated in this study.

### 3.6 Phage Types and Phage Defense Systems in the Community‐Associated Cons

Phage therapy has emerged as a promising alternative to combat AMR in recent years, especially in infections caused by MDR bacteria including CoNS, for which treatment options are limited (Oliveira et al. 2019; Göller et al. 2021). Thus, characterizing the types of prophages which has successfully integrated into the host CoNS chromosome will enable us to identify phages that can be potentially activated under specific conditions. In our collection of six CoNS strains, phages were discovered exclusively in *S. capitis* subsp. *urealyticus* (i.e., strains E_e2 and T_5b) with none detected in the three *S. epidermidis* strains. Two types of temperate siphoviruses were identified, namely StB12 and CNPx (Figure 4), both of which are known to infect various *Staphylococcus* species (Oliveira et al. 2019; Chou‐Zheng and Hatoum‐Aslan 2022; Verbanic et al. 2022; Lopes et al. 2025). Intact copies of both phage types were present in the E_e2 genome, whereas only StB12 was found in T_5b (Figure 4). The StB12 phage is known as a member of the B10 subcluster, the largest within siphovirus cluster B, a group previously reported in multiple staphylococci, including *S. capitis* (Oliveira et al. 2019). This phage type was also classified under the *Azeredovirinae* subfamily and encodes lysogeny‐related proteins (Lopes et al. 2025) although it retains the capacity to trigger bacterial cell death and virion release upon induction. Similarly, the CNPx phage exhibits a lysogenic life cycle and comparable functionality (Verbanic et al. 2022; Zhang 2025). CNPx is a member of the *Rockefellervirus* subfamily (Lopes et al. 2025) but has not yet been assigned to a specific cluster. To date, CNPx phages have been described primarily in *S. epidermidis* hosts and have been associated with the healing of chronic wounds (Depardieu et al. 2016; Chou‐Zheng and Hatoum‐Aslan 2022; Verbanic et al. 2022; Zhang 2025) although no phages were found in our three *S. epidermidis* strains. The finding of the CNPx phage in *S. capitis* subsp. *urealyticus* expands the known reservoir of these phages beyond *S. epidermidis* and adds to our catalog of phages from community‐associated CoNS, warranting further investigations regarding their functionality.

Bacteria have also developed their own antiphage mechanisms, or phage defense systems, to protect from viral invasions (Georjon and Bernheim 2023; Hossain et al. 2024). Figure 4 indicated that the *S. capitis* and *S. epidermidis* strains in this study harbor different repertoires of phage defense systems. The three *S. epidermidis* strains possess seven types of defense systems, encompassing Type_I_S51, Type_II_REase_34, Type_II_Mtases_FAM_22, Type_I_Mtases_FAM_10, Type_I_Mtases_FAM_0_einsi_trimmed, GajA, and GajB_1, and these defense systems hardly overlap with those found in *S. capitis* (Figure 4). Of note, the Gabija phage defense system, made up of the GajA and GajB proteins, was found exclusively in the three *S. epidermidis* strains. Gabija functions through an abortive infection mechanism where the infected host cell is self‐induced to be killed to prevent the phage from replicating and spreading to other cells (Huo et al. 2024). Among the *S. capitis* strains (Figure 4), S*. capitis* subsp. *urealyticus* E_e2 possessed the highest number of defense systems (*n* = 9) as compared to the other two strains (*n* = 3 each for T_3b and T_5b).

The defense system *gcu233*, also known as gene cassette of unknown function, was found in all the three *S. capitis* genomes (Figure 4). Although the actual mechanism of *gcu233* in phage defense awaits experimental validation, other variants of *gcu* (such as *gcu22* and *gcu23*) have been found to enable some levels of phage resistance (Kieffer et al. 2024). Several restriction modification (RM) systems were identified from *S. capitis* subsp. *urealyticus* (Figure 4); the RM type I‐III systems function to protect host DNA through sequence motif modification mediated by their corresponding methyltransferase (MTase), preventing host DNA cleavage by restriction endonuclease (REase) during phage invasion (Birkholz et al. 2022; Takahashi et al. 2025). Different from types I‐III, the RM type IV system functions to recognize methylated foreign DNA (i.e., phage) and cleaves it using the REase encoded by the system (Birkholz et al. 2022; Takahashi et al. 2025). A type IV RM system, denoted as RM_Type_IV_FAM_1, was detected in both *S. capitis* subsp. *urealyticus* E_e2 and T_5b but not in *S. capitis* subsp. *capitis* T_3b (Figure 4). This system was recently studied in other bacteria, such as *Helicobacter pylori*, *Enterococcus faecalis*, and *Pectobacterium carotovorum*, and demonstrated antiphage properties through the production of its REase (Birkholz et al. 2022; Bullen et al. 2024; Takahashi et al. 2025). Types I and II RM systems were also identified, but these were only found in *S. capitis* subsp. *urealyticus* E_e2 (Figure 4). Interestingly, CRISPR‐Cas systems were not identified in either of the *S. capitis* or the *S. epidermidis* strains. Since the presence of a CRISPR‐Cas system, specifically CRISPR‐Cas Type III, is one of the markers of the *S. capitis* NRCS‐A clone (Felgate et al. 2023), its absence indicates that E_e2 is possibly a proto‐NRCS‐A clone instead.

### 3.7 Core Genome Phylogeny of the Community‐Associated *S. capitis* and *S. epidermidis* Strains

SCC*mec* typing, AMR and virulome profiles (see sections above) indicate that *S. capitis* subsp. *urealyticus* strain E_e2 bears the characteristic hallmarks of the epidemic *S. capitis* NRCS‐A clone. In particular, the presence of the composite SCC*mec*‐SCC*cad*‐SCC*ars*‐SCC*cop* cassette (Figure 2), an MDR profile (Figure 3) and detection of NRCS‐A signature virulence genes (Figure 4) (Felgate et al. 2023) classifies E_e2 as an NRCS‐A clone. While NRCS‐A isolates that have been described were predominantly of clinical origin (Simões et al. 2016; Laurent and Butin 2019; Wirth et al. 2020; Wan et al. 2023; Crepin et al. 2024), E_e2 represents a community‐originated NRCS‐A strain.

**Figure 5:**
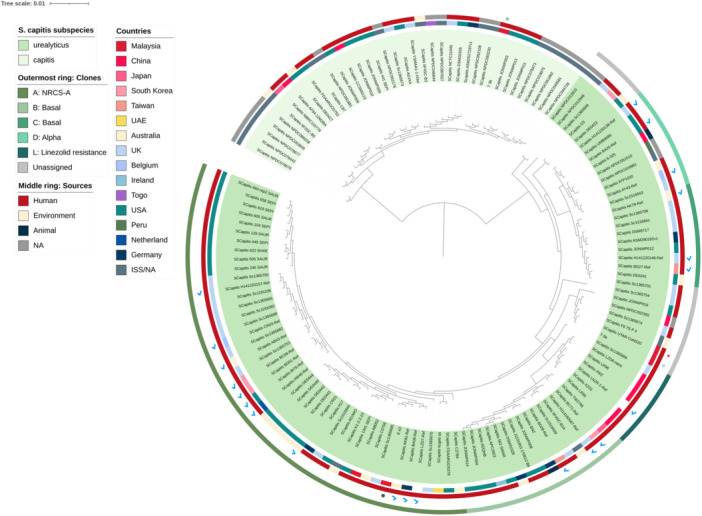
Core‐genome maximum‐likelihood phylogenetic analysis of *S. capitis* genomes, encompassing the subspecies urealyticus and capitis. Strains from this study were indicated with colored dots in the second outermost ring, UTAR‐CoNS20 from our previous study (Lean et al. 2025) was marked with red star in the same ring. Known references of *S. capitis* subsp. *urealyticus* lineages (i.e., clades A, B, C, D, and L; [Wang et al. 2022]) were indicated in blue ticks.

Core genome analysis places strain E_e2 within the NRCS‐A cluster (Figure 5), phylogenetically adjacent to the proto‐outbreak I (also known as clade A1) *S. capitis* strains AK81, BA08 and LZD7 (Wirth et al. 2020; Wang et al. 2022; Wan et al. 2023). Notably, these reference strains harbor SCC*mec* type IV (Wang et al. 2022), which aligns with the identification of the novel composite SCC*mec* homologous to SCC*mec* IVa/IVn in strain E_e2 (see SCC*mec* section above). Furthermore, the absence of a CRISPR‐Cas system in E_e2 is also indicative of its proto‐NRCS‐A lineage (Felgate et al. 2023). Conversely, the *S. capitis* subsp. *urealyticus* strain T_5b clustered outside the NRCS‐A lineage (Figure 5), and grouped closely with strain UTAR‐CoNS20 (Figure 5) from our previous study (Lean et al. 2025). As both strains (i.e., T_5b and UTAR‐CoNS20) were isolated from the same university campus (Universiti Tunku Abdul Rahman, Sungai Long Campus, Malaysia), their close phylogenetic relationship (with ANI values of 99.93%) likely reflects the convergence of microbiota among individuals in shared environments (Bewick et al. 2019). Nevertheless, these were not identical isolates, as supported by their different SCC cassette composition (Lean et al. 2025). Finally, strain T_3b clustered within the *S. capitis* subsp. *capitis* clade (Figure 5), which validates its taxonomic identity.

**Figure 6:**
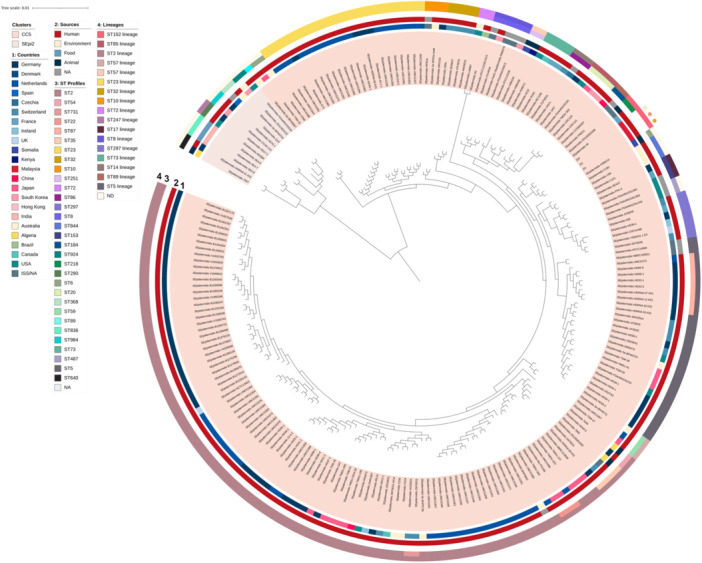
Core genome maximum‐likelihood phylogenetic tree of *S. epidermidis* genomes, labeled with ST lineages known till date. Colors of lineages (i.e., ring 4 from the center) were based on the assigned ST (i.e., ring 3 from the center), whereby lineage color was taken from the ST if it was the founder ST. For example, the ST23 lineage (ring 4) was colored yellow due to the founder ST23 (ring 3). The *S. epidermidis* strains from the current study were marked with colored dots on the outermost ring, and map alongside ST218 isolates, forming a distinct subclade within this background. Reference *S. epidermidis* genomes were taken from previously established lineages (Miragaia et al. 2007; Rolo et al. 2012; Lee et al. 2018).

The *S. epidermidis* strains ZG, ZH and ZW were assigned to the novel sequence type ST1284 (Table 1), which is a single locus variant of ST218, indicating recent diversification from this background. In the core‐genome phylogeny, ZG, ZH and ZW map alongside ST218 isolates, forming a distinct subclade (Figure 6), consistent with recovery of a closely related lineage from the same symptomatic ocular episode. ST218 *S. epidermidis* has been detected in both clinical and non‐clinical sources, including community carriage and animals, with isolates often being methicillin susceptible (Du et al. 2013; Arora et al. 2020; Persson Waller et al. 2025). This aligns with our findings as strains ZG, ZH, and ZW were also identified as methicillin‐susceptible *S. epidermidis*.

Beyond core‐genome phylogeny placement, the accessory genome profiles of the *S. epidermidis* strains were also broadly congruent. The three *S. epidermidis* genomes contained relatively fewer VFs compared with the *S. capitis* strains, and with largely overlapping repertoires dominated by surface‐associated proteins, exoenzymes and the *hld‐*encoded pro‐inflammatory peptide (Section 3.5, Figure 4). No SCC elements or prophages were detected in any of the three *S. epidermidis* genomes, and each strain harbored two plasmids—one which is 1.3 kb in size and the other one larger at 17.4 kb (see following Section 3.8). These findings collectively contextualize ST1284 as a methicillin‐susceptible GC1 *S. epidermidis* lineage with a conserved accessory‐genome profile in this case study. As *S. epidermidis* is a common commensal and opportunist, recovery from a symptomatic site should be interpreted cautiously and does not, on its own, establish causality.

The majority of currently sequenced *S. epidermidis* genomes are dominated by the ST2 lineage (Figure 6), largely due to sampling bias towards clinical isolates (Lee at al. 2018). The population structure of *S. epidermidis* as assessed by MLST, comprises six genetic clusters (GCs) (Thomas et al. 2014) with isolates from GC5 (comprising ST2 and ST23) predominantly nosocomial (Tolo et al. 2016). ST218, and by extension, ST1284, is categorized as GC1 (Tolo et al. 2016), and the detailed characterization of these less predominant lineages provides complementary perspectives on the ecology and spread of *S. epidermidis* which has so far been dominated by ST2 (Miragaia et al. 2007; Rolo et al. 2012; Du et al. 2013; Côrtes et al. 2022; Arora et al. 2020). For instance, the discovery of the global dissemination of the *S. epidermidis* ST23 lineage by Lee et al. (2018) highlighted the largely independent co‐evolution of ST2 and ST23 lineages in hospital settings. In the present study, the placement of ST1284 within GC1, together with its methicillin‐susceptible profile, is more consistent with a non‐nosocomial background that with the canonical hospital‐adapted ST2/GC5 lineages, although broader sampling would be required to confirm its prevalence or transmission dynamics.

### 3.8 Plasmids of the *S. capitis* and *S. epidermidis* Strains: Only the Small Plasmid of *S. capitis* subsp. *urealyticus* E_e2 Harbored an AMR Gene

**Figure 7:**
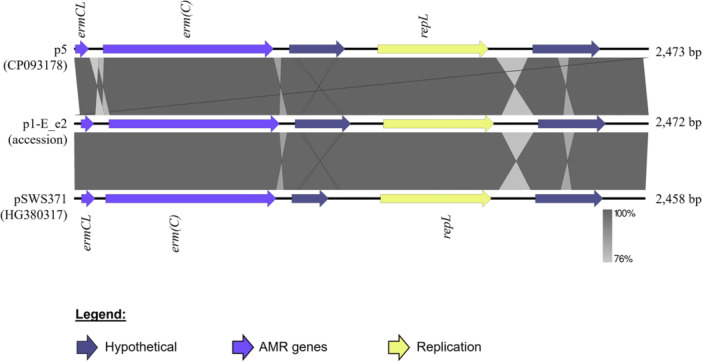
Plasmid map of p1‐E_e2 from *S. capitis* subsp. *urealyticus* E_e2 in comparison with similar plasmids p5 from *S. epidermidis* and pSWS371 from *S. aureus*. These plasmids harbor the *ermC* gene that mediates erythromycin resistance and its upstream *ermCL* that encodes the *ermC* leader peptide enabling regulation by attenuation, thereby yielding the inducible erythromycin resistance (or iMLS_B_) phenotype in their hosts.

Plasmids serve as key mobile genetic elements that facilitate the acquisition and transmission of AMR determinants between closely related bacteria. Among the *S. capitis* and *S. epidermidis* strains analyzed, only *S. capitis* subsp. *urealyticus* E_e2 harbored a plasmid‐borne AMR gene, *ermC*. The gene was encoded on a small 2,473 bp plasmid designated p1‐E_e2 (Figure 7). Sequence analysis revealed the presence of the leader peptide gene, *ermCL*, upstream of *ermC*. This arrangement allows for the regulation of *ermC* expression via translational attenuation, resulting in an inducible MLS_B_ (iMLS_B_) phenotype (Catchpole et al. 1988; Lüthje and Schwarz 2007; Al‐Trad et al. 2023), which was phenotypically validated using the D‐test. As shown in

Figure 7, p1‐E_e2 is nearly identical to the common small staphylococcal plasmids that are typically between 2.3 and 2.5 kb in size (Feßler et al. 2018) found in species such as *S. epidermidis* (e.g., the p5 plasmid; accession no. CP093178) and *S. aureus* (e.g., pSWS371; accession no. HG380317). This category of plasmids is known to carry the plasmid replication gene *repL* alongside *ermC* (Catchpole et al. 1988; Feßler et al. 2018). Strain E_e2 also harbored a second, larger plasmid (p2‐E_e2; of 5948 bp) which appeared cryptic. While the two other *S. capitis* strains, T_3b and T_5b, also harbored plasmids of varying sizes (Table 1), none encoded AMR determinants.

Similarly, the *S. epidermidis* strains ZG, ZH, and ZW each carried two plasmids, neither of which harbored AMR genes (Figure A1). The first group of plasmids (p1‐ZG, p1‐ZH and p1‐ZW) were very small plasmids of only 1,318 bp and encoded only the plasmid replication gene along with a hypothetical open reading frame (ORF) (Figure A2). The second group (p2‐ZG, p2‐ZH and p2‐ZW) was approximately 17.4 kb in size and carried genes facilitating plasmid mobilization (Feßler et al. 2018).

## 4 Conclusions

The CoNS have previously been regarded as commensals, yet they can act as opportunistic pathogens and their potential impact on health should not be underestimated. As highlighted by Lee et al. (2018), *S. epidermidis* was routinely excluded from routine screening in hospitals for this reason, and greater vigilance towards CoNS was thus recommended. The growing number of recent reports on CoNS, particularly the *S. capitis* NRCS‐A clone (Wang et al. 2022; Wan et al. 2023; Crepin et al. 2024), has drawn attention due to their pathogenicity. However, these reports raised unresolved questions on the source and routes of dissemination of these pathogens.

In this context, the detection and genomic characterization of *S. capitis* in this study, in particular the identification of *S. capitis* subsp. *urealyticus* E_e2 as a proto‐outbreak I (proto‐NRCS‐A) clone from the infected ear of an otherwise healthy individual, suggests that healthcare‐associated lineages can be encountered outside of healthcare settings. However, it should be noted that this observation is based on a single case and therefore does not establish population‐level community circulation, transmission routes, or a definitive reservoir. Nevertheless, it supports the rationale for broader surveillance to determine how frequently NCRS‐A‐like lineages occur in community carriage and under what environmental or clinical conditions they may emerge. Consistent with the individual's lack of prior healthcare‐associated antibiotic selection pressure, E_e2 remained vancomycin‐susceptible (Figure 3). While the strain recovered here was not associated with severe disease, lineages of similar genetic background could become clinically consequential if introduced into healthcare environments where selection and transmission pressures differ.

Skin microbiota within a specific community, especially in settings where individuals coexist or interact in close proximity, tend to converge even among unrelated individuals (Bewick et al. 2019; Boxberger et al. 2021). The observed genetic relatedness between *S. capitis* subsp. *urealyticus* T_5b (from this study) and UTAR‐CoNS20 (obtained from a nasal swab of a previous study in 2024; [Lean et al. 2025]), is an example of this correlation among community‐derived CoNS. The hosts of these two strains were complete strangers within the UTAR campus community, but they do share the same environment. Movement of people around this shared space likely facilitated an indirect exchange of bacteria, making it plausible for CoNS and other species to circulate without direct physical contact. This phenomenon underscores the community as a dynamic potpourri and reservoir of microbiota and highlights the potential for closed‐loop transmission where strains circulate between individuals and persist over time. Nevertheless, the extent, directionality, and frequency of such exchange cannot be hypothesized and resolved from the current dataset.

From birth, infants are exposed to multiple sources of microbiota (e.g., their mothers, caregivers, household contacts, and the surrounding environment) which may shape early colonization patterns and influence later carriage (Datta et al. 2021). Each individual can thus be considered a “vessel” for numerous bacteria. Under selection pressures imposed by lifestyle factors such as hygiene practices and antibiotic use, these bacteria adapt to survive the harsh and challenging environment of the skin surface. The coexistence of *S. capitis* (strains E_e2, T_3b, and T_5b) and *S. epidermidis* (strains ZG, ZH, and ZW) in this study not only hints at dominating CoNS species on a healthy individual, but also illustrates the shifts in bacterial communities under specific conditions. Longitudinal sampling enabled us to document the changes in *S. capitis* carriage during and after an ear infection, including the recovery of different *S. capitis* subspecies (*S. capitis* subsp. *urealyticus* E_e2 during symptoms and strains T_3b and T/5_b post‐recovery). We also recovered *S. epidermidis* isolates (ZG, ZH, and ZW) during a subsequent symptomatic ocular episode, highlighting site‐ and episode‐specific variability within common CoNS strains. These findings are best interpreted as illustrating the dynamic nature of CoNS colonization and the opportunistic nature of particular lineages under perturbed conditions, rather than providing definitive evidence of causality for infection. Given that most epidermal infections are treated with topical antibiotics, careful consideration of antibiotics selected for treatment and awareness of selection pressure from antibiotic overuse are essential to prevent the emergence and enrichment of more harmful traits in otherwise commensal organisms.

This study has limitations, including sampling from a single individual and the inability to perform more extensive follow‐up sampling due to loss of contact. Participant withdrawal is an unavoidable aspect of human‐subject research. In addition, for the ear episode, only a single colony representing the predominant colony morphotype was selected for downstream characterization and WGS; minor morphotypes were not retained and hence, within‐sample strain diversity or mixed colonization during the infection episode cannot be excluded. Collectively, these constraints limit the generalizability and preclude population‐level inference. A more systematic sampling strategy involving larger cohorts with denser longitudinal sampling (and ideally, integrating culture‐independent profiling alongside isolate genomics) would be required to better understand CoNS transmission routes, persistence of colonization, and the extent to which healthcare‐associated lineages such as NRCS‐A circulate in the community.

## Author Contributions

**Soo Sum Lean:** writing – original draft (lead), formal analysis (lead), investigation (equal), methodology (equal), data curation (lead), software (lead), validation (equal), visualization (lead), writing – review and editing (equal). **Chew Chieng Yeo:** conceptualization (equal), writing – original draft (equal), formal analysis (equal), investigation (equal), validation (equal), writing – review and editing (equal). **Zain Illyaaseen:** conceptualization (equal), formal analysis (equal), investigation (equal), methodology (equal), writing – review and editing (equal). **Sargit Kaur:** conceptualization (equal), formal analysis (equal), investigation (equal), methodology (equal), writing – review and editing (equal). **Yun Fong Ngeow:** conceptualization (equal), funding acquisition (supporting), resources (supporting), writing – review and editing (equal). **Stuart C. Clarke:** writing – original draft (equal), resources (supporting), writing – review and editing (equal). **Hien Fuh Ng:** conceptualization (lead), writing – original draft (equal), funding acquisition (lead), investigation (lead), methodology (equal), data curation (equal), validation (equal), resources (lead), supervision (lead), writing – review and editing (equal).

## Ethics Statement

Informed consent was obtained from the patient, and the study was approved by the UTAR Scientific and Ethical Review Committee (U/SERC/78‐297/2024). All experiments were performed in accordance with relevant guidelines and regulations.

## Consent

All authors have provided consent for publication.

## Conflicts of Interest

The authors declare no conflicts of interest.

## Supporting information


**Figure A1:** Comparative genetic map of the composite SCC*mec* type IV identified from the *Staphylococcus capitis* subsp. *urealyticus* E_e2 genome with the SCC*mec* type V‐SCC*cad/ars/cop* composite found in the genome of *S. capitis* strain CR01 (accession no. KF049201) (Simões et al., 2014), along with the canonical SCC*mec* type IVa (accession no. AB063172) and SCC*mec* type IVn (accession no. KX385846.1). Grey‐shaded areas in between the linear maps of each SCC*mec* depict areas of DNA sequence identities as indicated by the vertical bar at the bottom right of the figure. **Figure A2:** Plasmids identified from *S. epidermidis* strains ZG, ZH, and ZW in this study. Blue‐colored thread and needle icons represent points whereby contigs that were identified as plasmid‐origin were patched together into a single scaffold based on homologous plasmids in the database.

**Table A1:** Antimicrobial susceptibility assay results for the six CoNS isolates in this study. Susceptibility was determined using the BD Phoenix M50 PMIC‐GPC panel to obtain MIC values and Kirby‐Bauer disk‐diffusion tests with zones of inhibition shown in mm.

## Data Availability

The genomes of the three *Staphylococcus capitis* and three *Staphylococcus epidermidis* isolates have been deposited in the National Center for Biotechnology Information (NCBI)'s Genomes database under BioProject No. PRJNA1377288.
