# Supplementary material for: Of Commensals and Opportunists: Genomics of Coagulase‐Negative Staphylococci During Sequential Ear and Eye Infections in a Healthy Adult
Source: Microbiologyopen. 2026 Mar 31;15(2):e70277. doi: 10.1002/mbo3.70277 (PMC13140555; doi:10.1002/mbo3.70277)
Supplement: Supplementary file 1 — Figure A1: Comparative genetic map of the composite SCCmec type IV identified from the Staphylococcus capitis subsp. urealyticus E_e2 genome with the SCCmec type V‐SCCcad/ars/cop composite found in the genome of S. capitis strain CR01 (accession no. KF049201) (Simões et al., 2014), along with the canonical SCCmec type IVa (accession no. AB063172) and SCCmec type IVn (accession no. KX385846.1). Grey‐shaded areas in between the linear maps of each SCCmec depict areas of DNA sequence identities as indicated by the vertical bar at the bottom right of the figure. Figure A2: Plasmids identified from S. epidermidis strains ZG, ZH, and ZW in this study. Blue‐colored thread and needle icons represent points whereby contigs that were identified as plasmid‐origin were patched together into a single scaffold based on homologous plasmids in the database. [file MBO3-15-e70277-s002.pdf]

# Of commensals and opportunists: Genomics of coagulase-negative staphylococci during sequential ear and eye infections in a healthy adult

Soo Sum Lean[1], Chew Chieng Yeo[2], Zain Illyaaseen[3], Sargit Kaur[3], Yun Fong Ngeow[3], Stuart C. Clarke[1,2,4,5,6*] and Hien Fuh Ng[3*]

## APPENDIX

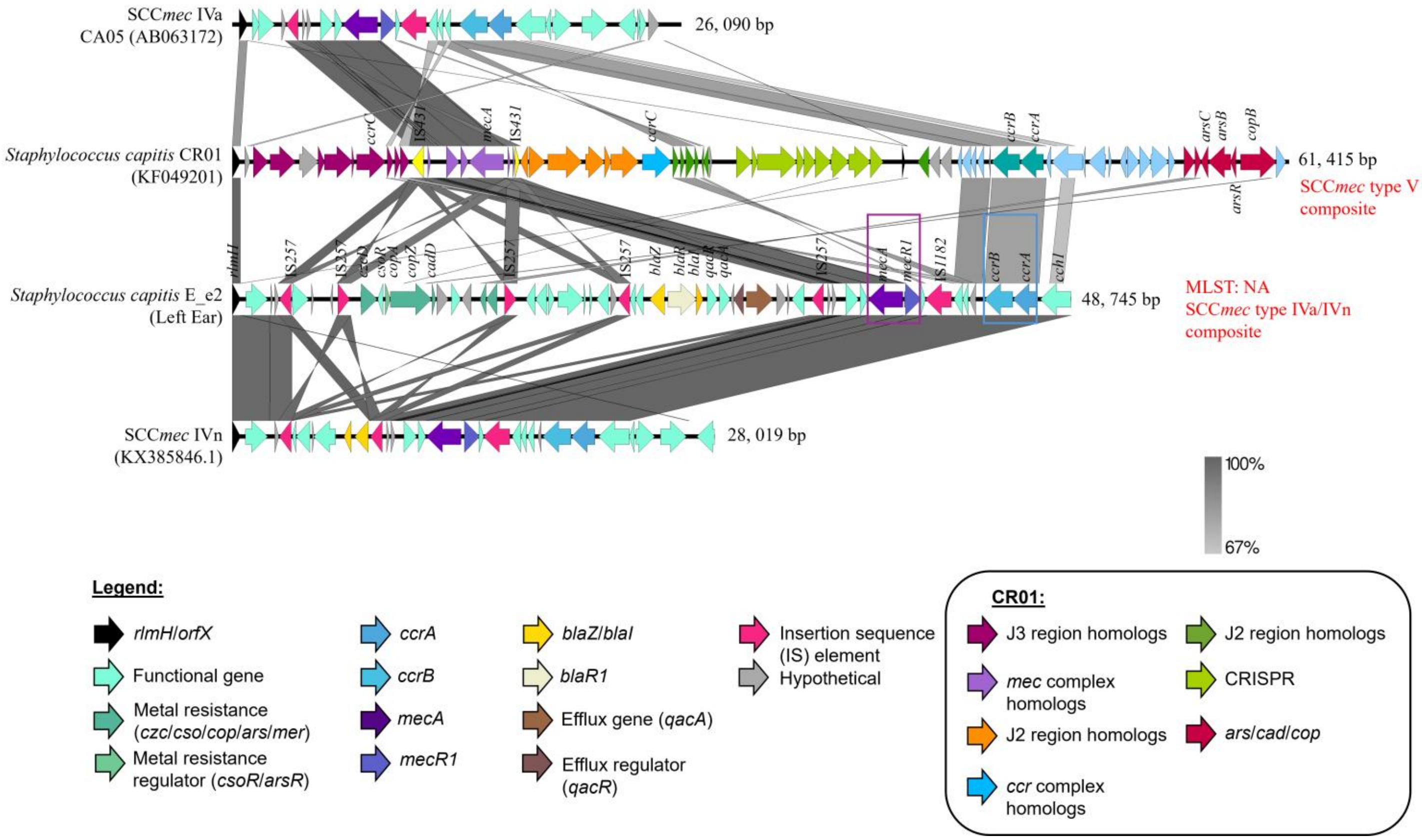


**Figure A1.** Comparative genetic map of the composite SCC*mec* type IV identified from the *Staphylococcus capitis* subsp. *urealyticus* E_e2 genome with the SCC*mec* type V-SCC*cad/ars/cop* composite found in the genome of *S. capitis* strain CR01 (accession no. KF049201) (Simões et al., 2014), along with the canonical SCC*mec* type IVa (accession no. AB063172) and SCC*mec* type IVn (accession no. KX385846.1). Grey-shaded areas in between the linear maps of each SCC*mec* depict areas of DNA sequence identities as indicated by the vertical bar at the bottom right of the figure.

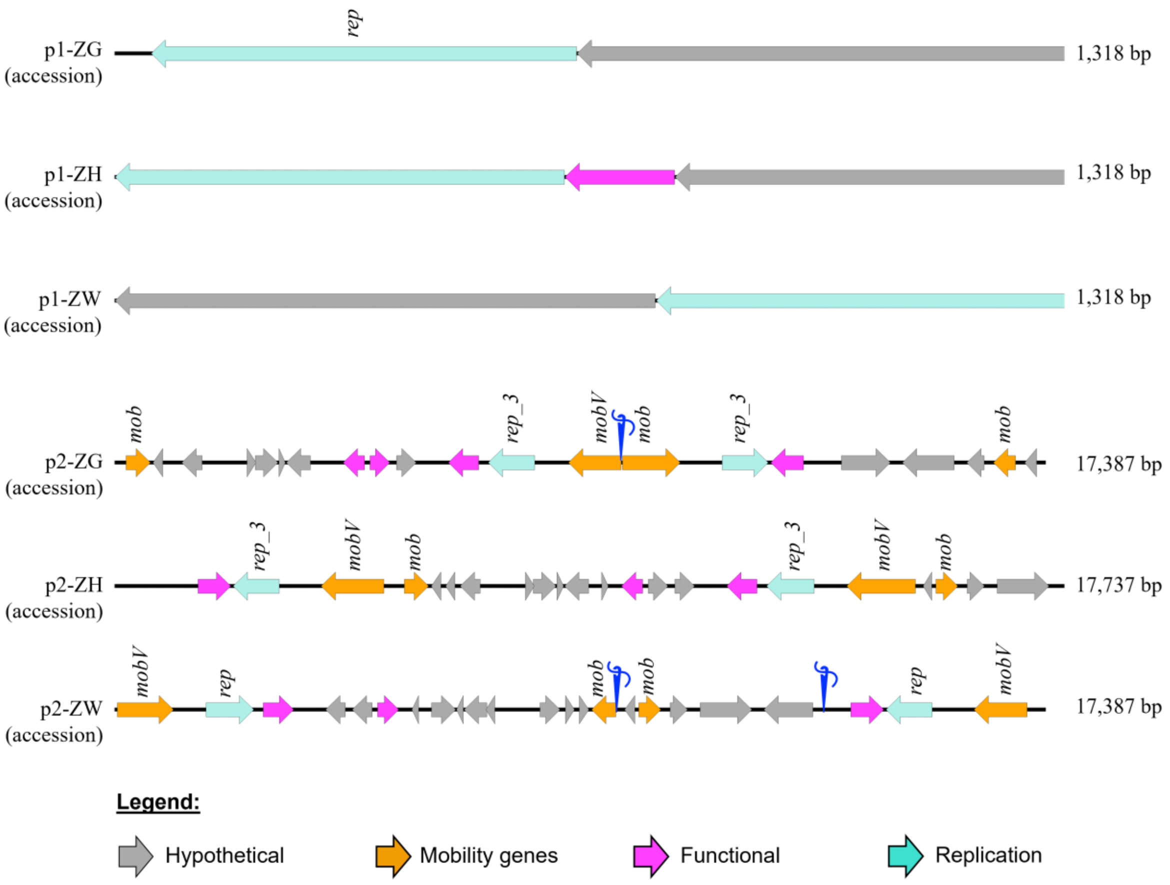


**Figure A2**: Plasmids identified from *S. epidermidis* strains ZG, ZH and ZW in this study. Blue-coloured thread and needle icons represent points whereby contigs that were identified as plasmid-origin were patched together into a single scaffold based on homologous plasmids in the database.
